# Assessment of the prevalence and associated risk factors of pediatric hydrocephalus in diagnostic centers in Addis Ababa, Ethiopia

**DOI:** 10.1186/s12887-022-03212-6

**Published:** 2022-03-18

**Authors:** Blein Mulugeta, Girma Seyoum, Abebe Mekonnen, Elbet Ketema

**Affiliations:** 1 Department of Medical Radiologic Technology, Addis Ababa University, Addis Ababa, Ethiopia; 2 Department of Anatomy, Addis Ababa University, Addis Ababa, Ethiopia; 3 Department of Radiology, Addis Ababa University, Addis Ababa, Ethiopia; 4 Departmetn of Pediatrics, Addis Ababa University, Addis Ababa, Ethiopia

**Keywords:** Cerebrospinal fluid, CT, Hydrocephalus, MRI and neural tube defects

## Abstract

**Background:**

Hydrocephalus (HCP) is a common disorder of cerebral spinal fluid (CSF) physiology resulting in abnormal expansion of the cerebral ventricles. Infants commonly present with progressive macrocephaly whereas children older than 2 years generally present with signs and symptoms of intracranial hypertension. Neither qualitatively nor quantitatively are there adequate data to determine the prevalence and incidence of HCP in the developing world. HCP is a treatable condition that when left untreated, has fatal consequences.

**Objective:**

The objective of this study was to assess the prevalence of pediatric HCP and associated risk factors in diagnostic centers in Addis Ababa, Ethiopia.

**Methods:**

This study was conducted using a cross-sectional facility-based study design over a two-time period, i.e. a 2-year retrospective data collection from January 2018 to January 2020 included 1101 patients and a prospective data collection from May 2019 to February 2020 included 99 patients. Children aged 5 years and below who came to the selected diagnostic centers for MRI/CT examination were studied. The collected data were analyzed using binary logistic regression.

**Result:**

The retrospective study included 639(58%) males and 462 (42%) females. The mean age calculated was 22.3 months. Infants aged younger than 24 months 753 (68.4%) were significantly associated with HCP development (*P* < 0.05). In the retrospective study, HCP etiologies; Aqueductal stenosis (17.9%), Neural Tube defects (NTDs) (35.7%), post-infectious (10.1%) were identified. In the prospective study, the gender and age distribution was 57(57.6%) males, 42 (42.4%) females, 60.6% infants aged younger than 24 months with a mean age of 24.9 months. Inadequate consumption of folic acid and development of HCP was found to be statistically significant (*P* < 0.05). In the prospective study, HCP etiologies; Aqueductal stenosis (26.1%), Neural Tube defects (26.08%), and post-infectious (8.69%) were identified. The 3 years prevalence of HCP calculated in both studies was 22% (223 per 1000 live births).

**Conclusion:**

The results of this study suggest that the high prevalence of HCP was due to the high prevalence of aqueductal stenosis and neural tube defects; with a small contribution of post-infectious causes. The majority of infants who present with HCP were aged younger than 24 months.

## Background

Hydrocephalus (HCP) is defined as a pathophysiology with disturbed cerebrospinal fluid (CSF) circulation [1]. Infants commonly present with progressive macrocephaly whereas children older than 2 years generally present with signs and symptoms of intracranial hypertension [2]. In the developed world, the incidence of congenital HCP has been estimated to be about 0.5 cases per 1000 live births and the overall incidence of neonatal HCP is estimated to be about 3 to 5 cases per 1000 live births [3]. Neither qualitatively nor quantitatively are there any adequate data/literature to determine the prevalence and incidence of HCP in the developing world, especially in sub-Saharan Africa, where this condition appears to be much more frequent than in developed countries. Taking some very conservative estimates in Uganda, as an example, between 1000 and 2000 new cases of infant HCP occurs every year, with this most likely being an underestimate. Extrapolating this to other regions suggests 6500 new cases per year in East Africa and more than 45,000 new cases per year in sub-Saharan Africa [4]. HCP is a common neurosurgical disorder that can lead to significant disability or death if not promptly identified and treated. Data on the burden of HCP in low-income countries are limited, given a lack of radiologic resources for the diagnosis of this condition [5]. In Ethiopia, the etiology and incidence rates of HCP can be assumed to correspond to what has been reported from other East African countries, where HCP has been estimated to have an infectious origin. Only a minute fraction of children with HCP in Ethiopia – a few hundred each year - are diagnosed and receive proper treatment for their HCP. This probably means a selection bias that makes it difficult or even impossible to determine or estimate the general characteristics of HCP in Ethiopia concerning etiology, type of HCP, gender, or age composition of the patients. If the estimates on new cases of pediatric HCP in Ethiopia are valid, these children represent an enormous challenge for the nation [6]. HCP has many causes. Congenital HCP, most commonly involving aqueduct stenosis, and HCP can also be acquired, mostly from pathological processes that affect subarachnoid space function [2]. Continued research and education are essential if a significant reduction in morbidity and mortality from this disease is to be accomplished [4]. Hence, this study has assessed the prevalence and possible risk factors of pediatric HCP from a large sample of CT and MRI scans of the head performed at selected diagnostic centers in Addis Ababa, Ethiopia, a low-income country in Africa. The findings of this study will give focus on the management of HCP, and will create awareness for the importance of prenatal care given to mothers during their pregnancy.

## Methods and materials

This study was performed in four of the diagnostic centers that work in close association with Addis Ababa University, Wudassie Diagnostic Center (WDC), Dr. Alia Diagnostic Center, Pioneer Diagnostic Center, and BMY Diagnostic Center; all chosen randomly. This study was conducted from January 2018 to February 2020. A cross-sectional facility-based study was conducted over a two-time period, i.e. a 2-year retrospective data collection from January 2018 to January 2020 and a prospective data collection from May 2019 to February 2020. The study design involved children aged 5 years and below who came to the diagnostic centers for MRI/CT examination during the data collection periods. The retrospective data collection included 1101 patients and the prospective data collection included 99 patients. All children of either gender aged 5 years and below sent for Brain MRI /CT examination were included in the study. Patients, who had surgery for HCP before the study period started, patients who had imaging examinations besides the Brain, and parents/caretakers not willing to participate in the study were excluded. The retrospective data were collected by the employment of a checklist that included the patient’s clinical indication, the type of modality used, confirmation of HCP, and type of HCP confirmed. The prospective data were collected through a pre-tested semi-structured questionnaire that consisted of socio-demographic and economic factors, maternal conditions, and radiological results of the study participants. The data were entered, cleaned, and analyzed using SPSS software version 23.0. The data collected was analyzed by descriptive analysis. The 3 years prevalence of HCP was calculated in both the retrospective and prospective studies as the total included patients per 1000 live births). Bivariate analysis, using crudes odds ratio (COR), was used to test the degree of association between dependent and independent variables. Those variables that were identified to have a *P*-value less than 0.2 were included in the multivariate analysis, using adjusted odds ratio (AOR), to identify predictors of the outcome variable in the prospective study. These variables were; age, prenatal care, folic acid supplementation, family history of HCP, educational level of the mother, maternal pathologies, and infections. In the retrospective study, only multivariate analysis was applied because of the limited amount of variables. This was due to a lack of patient charts in diagnostic centers resulting in fewer clinical data. Both studies used a 95% confidence interval (CI) with a *P*-value of less than 0.05 taken as statistically significant. Ethical clearance was obtained from the Anatomy Department, College of Health Sciences, and the office of diagnostic centers. The purpose and importance of the study were explained to each study participant and they were informed that no personal identifiers were used in the data. Verbal and written consent was also obtained from each participant. Participants had the right to be excluded from the study if they were not voluntary to participate.

## Results

### Characteristics of study participants

In the retrospective study, a total of 1101 children under 5 years of age participated. Of these, 639(58%) were males and 462(42%) were females. The mean age and standard deviations were 22.3 months and ± 18.8 respectively. There were 753 (68.4%) children aged younger than 24 months. The number of children diagnosed with HCP during the retrospective study was 245 (22.3%), of these HCP cases, 153(62.4%) were classified as non-communicating HCP. The proportion of males diagnosed with HCP was relatively higher. Table 1.

**Table 1:** Characteristics of Study Participants of the Retrospective Study within the Selected Diagnostic Centers in Addis Ababa, Ethiopia, 2018-2020

Variable	Category	Frequency	Percent (%)
Age of child	≤24 months	753	68.4
Gender	Male	639	58
	Female	462	42
Type of modality used	MRI	736	66.8
HCP diagnosed cases	Yes	245	22.3
HCP Type	Non-communicating	153	62.4
HCP diagnosed cases	In males	140	57.14

Similarly, children under 5 years of age were included in the prospective data with a mean age of 24.9 months and a standard deviation of ±19.2. Of a total of 99 children, 57 (57.6%) were males and 42(42.4%) were females. Ninety-three (93.9) were imaged using MRI. This data set had 60 (60.6%) children aged younger than 24 months. Of the number of children diagnosed with HCP 23 (23.2%); 13(56.5%) were classified as non-communicating HCP. The proportion of females diagnosed with HCP was relatively higher in this study Table 2. The history of HCP among families was found to account for 46 (46.5%) of all the interviewed cases. Also, it was identified that 55.6% of the families had a first-degree relative. Regarding the maternal history, occupational and educational status are all described in Table 2.

**Table 2:** Characteristics of Study Participants of the Prospective Study within the Selected Diagnostic Centers in Addis Ababa, Ethiopia, 2019-2020

Variables	Category	Frequency	Percent (%)
Age of child	≤24 months	60	60.6
HCP diagnosed cases	Yes	23	23.2
HCP Type	Non-communicating	13	56.5
HCP diagnosed cases	In females	12	57.12
Age of mother	18-23	23	23.7
	24-29	45	46.4
	29-34	17	17.5
	35-40	12	12.4
	Did not attend school	22	24.2
The educational level of the mother	Elementary school(1-8)	29	31.9
	High school(9-12)	22	24.2
	Diploma and above	18	19.8
Occupational status of the mother	Employed	17	18.7
	Housewife	74	81.3
Type of modality used	MRI	93	93.9
Residential area	Urban	50	50.5
	Rural	49	49.5
Antenatal care follow up	Yes	75	82.4
Mode of delivery	Cesarean	19	20.9
	Vaginal	71	78
Trauma during pregnancy	Yes	8	8.8
Pre-eclampsia	Yes	9	9.9
Sexually transmitted disease	Yes	4	4.4
Diabetes mellitus	Yes	2	2.2
Uterine infection	Yes	9	9.9
Folic acid supplement	Yes	61	67.8
Usage of folic acid	Before conceiving	3	3.3
	After conceiving	58	58.6
History of HCP	Yes	46	46.5
Family relatives	First degree	5	55.6
	Second degree	2	22.2
	Third degree	2	22.2

### Prevalence of HCP

The number of children diagnosed with HCP in the 3 years in both retrospective and prospective studies was 22.3% i.e. 222.72 per 1000 births (2222.72 per 10,000 births).

### Prevalence of congenital and acquired HCP

**Fig. 1:**
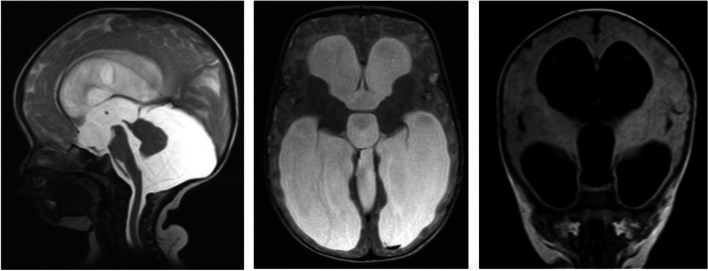
Brain MRI of a 4 months old male infant. Clinical indication: Hydrocephalus. Description: Sagittal T2, Axial fast spin echo (FSE T2), and Coronal Fluid attenuation inversion recovery (FLAIR) sequences performed on a 1.5 T MR scanner revealed grossly dilated lateral and third ventricles with posterior fossa cyst communicating with the fourth ventricle inferiorly and hypoplastic cerebellar vermis. The cerebellar hemispheres are also small and displaced anteriorly. The surrounding cerebral parenchyma is thinned out. The major intracranial arterial and venous flow-voids are patent. The visualized portions of the paranasal sinuses and orbits are unremarkable. IMPRESSION: Hydrocephalus with posterior fossa cyst and cerebellar vermis hypoplasia likely Dandy-Walker malformation

Prevalence of congenital and acquired HCP in both study periods was studied based on MRI and CT images, of all children diagnosed with HCP (22.3%). Congenital HCP secondary to Aqudectal stenosis, 17.6 and 26.1% in the retrospective and prospective study respectively, was identified to be the most prevalent. (Fig. 1). Neural tube defects (NTDs) were identified as the second most prevalent cause. Chari II malformation and Dandy-Walker variant account for the majority of NTDs identified in the retrospective and prospective study respectively. (Tables 3 and 4). Acquired HCP secondary to post-infectious, post-hemorrhagic, and tumor-related causes was identified. Post-infectious, 10.2 and 8.69% identified in retrospective and prospective periods respectively was attributed to post-meningitis complications and chronic in-utero infection (TORCH- Toxoplasmosis, Rubella, Cytomegalovirus, and Herpes), Ventriculitis, Cerebral and Cerebellar abscess (Tables 3 and 4).

**Table 3:** Congenital and Acquired Pediatric HCP- Retrospective data compilation from the Selected Diagnostic Centers in Addis Ababa, Ethiopia, 2018-2020

Congenital and Acquired HCP	***n***	%
Aqueductal Stenosis	44	17.9
Chari II malformation	41	16.7
Dandy-Walker Malformation	28	11.4
Colpocephaly	8	3.2
Encephalocele	5	2.04
Myelomeningocele	2	0.81
Meningoencephalocele	3	1.2
Post-infectious	8	3.2
Post-meningitis	17	6.9
Adhesion of Foramen of Monroe	1	0.4
Medulloblastoma	12	4.8
Pineal gland glioma	1	0.4
Ependymoma	1	0.4
Craniopharyngioma	2	0.8
Subdural Hematoma	1	0.4
Hypoxic-ischemic cerebral injury	3	1.2
Perinatal ischemic insult	3	1.2
Cystic Encephalomalacia	2	0.8
Dural venous malformation	1	0.4

**Table 4:** Prevalence of Congenital and Acquired Pediatric HCP- Prospective data compilation from the Selected Diagnostic Centers in Addis Ababa, Ethiopia, 2019-2020

Congenital and Acquired Pediatric HCP	***n***	%
Aqueductal Stenosis	6	26.1
Dandy-Walker malformation	4	17.4
Chari II Malformation	2	8.7
Meningocele	2	8.69
Meningoencephalocele	2	8.69
Post-meningitis	2	8.69
Medulloblastoma	2	8.69
Germinoma	2	8.69
Craniopharyngioma	2	8.69
Subdural Hematoma	5	21.7
Ex-vacuo ventriculomegaly due to Post-perinatal hypoxic-ischemic cerebral injury	5	21.7
Ex-vacuo ventriculomegaly due to perinatal hypoxic-ischemic cerebral injury	3	13.04

### Risk factors associated with HCP

The retrospective study found that those children aged under 24 months diagnosed with HCP 192(78.3%) had a 1.9 times higher risk of developing HCP than those aged above 24 months which was found to be statistically significant; (AOR = 1.90 [95% CI = 1.36, 2.26], *P* < 0.05). Table 5.

**Table 5:** Associated Risk factors of HCP of the Retrospective Data within the Selected Diagnostic Centers in Addis Ababa, Ethiopia, 2018-2020

Variables	Category	Diagnosis		COR (CI 95%)	AOR (CI 95%)	***P***-value
		Yes	No			
Age of the child	≤24 months	192	559	1.89 (1.36, 2.65)*	1.90 (1.36, 2.26)^*^	0.000

Note: ^*^significance; *p* < 0.05

In the prospective study, the number of children aged under 24 months diagnosed with HCP was 15 (65.2%). Even though this accounted for the majority of the HCP patients, it was not statistically significant (*P* > 0.05). Gender also was not significantly associated with the presence of HCP (*P* > 0.05). Table 6. Mothers who did not take folic acid nutritional supplements 29 (32.8%) were identified to be significantly associated with HCP occurrence (AOR = 6.107 [95% CI = 1.32, 28.35], *P* < 0.05) Table 6. This study identified that interviewed mothers who had Pre-eclampsia were (9.9%), chronic hypertension (8.8%), and Diabetes mellitus (2.2%) during their gestation as potential risk factors but a significant association with HCP was not found (*P* > 0.05). No association (*P* > 0.05) was found with regards to advanced maternal age, family history of HCP, sexually transmitted infections, alcohol, and cigarette smoking among interviewed mothers. (Table 6).

**Table 6:** Associated Risk factors of HCP of the Prospective data within the Selected Diagnostic Centers in Addis Ababa, Ethiopia, 2019-2020

Variables	Category	Diagnosis		COR (CI 95%)	AOR (CI 95%)	***P***-value
		Yes	No			
Age of child	≥24 months	8	31	1	1	
	≤24 months	15	45	1.2(0.08 -1.3)	1.3 (0.6- 1.7)	0.606
Gender	Male	11	46	1	1	
	Female	12	30	0.59(0.23- 1.52)	1.69 (0.63- 4.54)	0.283
Age of mother	18-23	2	21	1	1	
	24-29	14	31	0.21(0.04- 1.03)	0.22 (0.04- 1.08)	0.054
	29-34	5	12	0.23 (0.038- 1.36)	0.24 (0.04- 1.43)	0.105
	35-40	2	10	0.48(0.06-3.88)	0.43 (0.05- 3.58)	0.489
The educational level of the mother	Did not attend school	8	14	1	1	
	Elementary school(1-8)	6	23	2.2(0.63-7.64)	2.04 (0.54- 7.79)	0.219
	High school(9-12)	4	18	2.6(0.64-10.31)	2.61 (0.57- 11.7)	0.183
	Diploma and above	3	15	2.8(0.63- 12.9)	3.92 (0.78- 19.5)	0.174
Antenatal care attendance	Yes	19	56	1	1	
	No	2	14	2.38(0.49- 11.41)	0.34 (0.06- 1.78)	0.280
Consumption of folic acid	Yes	19	42	1	1	
	No	2	27	6.107(1.32- 28.35)*	1.64 (0.052- 51.5)*	0.021*
Use of folic acid	Before conceiving	2	1	1	1	
	After conceiving	17	41	0.59(0.04-0.804)	0.27 (0.009- 8.32)	0.036*
History of HCP in the family	Yes	17	29	1	1	
	No	6	47	4.52 (1.624- 12.984)*	4.89 (0.64- 3.23)*	0.907
Trauma to the mother during pregnancy	Yes	4	4	1	1	
	No	17	66	3.882(0.88-17.140)	2.93 (0.56- 15.3)	0.073

Note: ^*^significance; *P* < 0.05

## Discussions

### Demographics: sex and age

As opposed to their female counterparts, males had a relatively higher chance of being diagnosed with HCP. This finding is similar to published studies in other African countries which have shown that the majority of pediatric patients with HCP are males; 64.6% of the population in a study from Tanzania and 53% in a study from Kenya [7]; 60.5% in a study from Nigeria [8], as well as a predominance of 51.9% in a study from Uganda [9], and 66.7% in another study in Ethiopia [6]. However, the prospective study had a different finding; as it was found that females had a relatively higher chance of being diagnosed with HCP. In this study, sex was not considered as one of the risk factors of HCP. As a result, the relatively higher prevalence of HCP among males in the retrospective study and females in the prospective study was not further investigated. In this study, there was a slight increment in the mean age of pediatric patients from 22.3 months in the retrospective study to 24.9 months in the prospective study but the difference was not statistically significant. A significant association between age and the development of HCP was found in the retrospective study, as children aged younger than 24 months had a higher risk of developing HCP similar to other published articles [10, 11]. Non-communicating HCP (obstructive HCP) predominance has been attributed to Aqueductal stenosis and NTDs in agreement with a similarly conducted Ethiopian study [6].

### Prevalence and etiologies of HCP

In developed countries, the incidence of congenital HCP is estimated at three to five cases per 1000 live births [3]. Contrary to this, the prevalence of HCP in this study in the 3 years was 22% (223 per 1000 live births) higher than prevalence rates seen in four European regions [12], Denmark [13], California [14], northern China [15], and prevalence in a Nigerian region [16]. However, the results of this study are in agreement with the study conducted in Uganda-from CURE Children’s Hospital of Uganda (CCHU) which demonstrated infant HCP prevalence rate between 1000 and 2000 cases every year [4]. This study is also similar to research conducted here in Addis Ababa, which used the estimates applied in the Ugandan research and presented an estimate of between 2000 and 4000 new cases of pediatric HCP per year [6]. In presenting the causes and incidence rates of HCP from Uganda [4], Tanzania [17], and Kenya [18] where it served as a reference for Ethiopia, there is a contradiction concerning the cause of HCP as a small contribution of post-infectious causes was found in this study. This discrepancy may be explained by differences in methodology. In the Ugandan study, for example, post-infectious HCP (PIH) was defined in terms of the absence of HCP at birth, history of febrile illness or seizure, and evidence of prior ventriculitis [4]. In this study, however, post-natal imaging investigations were studied thus defining PIH in terms of post-natal diagnosis. As described earlier, the high prevalence rate of HCP in this study has been attributed to high percentages of Aqueductal stenosis and NTDs similar to studies conducted in Zambia, Zimbabwe, and Malawi [19]. This study has also found HCP associated with meningomyelocele (MMC) similar to studies in Uganda [4], Kenya [18], Tanzania [17], south-western Saudi Arabia [20], and another Ethiopian study [6].

### The risk factors associated with pediatric HCP

It has been suggested that getting adequate prenatal care might help prevent birth defects, HCP being one of them [21]. Although an association between prenatal care given to mothers and HCP development was expected, this study did not find this to be significant, similar to a study from Mississippi [22]. It was initially hypothesized that mothers who attend their prenatal follow-ups would be aware of the use of folic acid thus avoiding NTDs that lead to HCP. This may be explained by the low literacy rate of female adults in Ethiopia that affects mothers’ ability to navigate the health care system. As previously predicted, due to the inadequate consumption of folic acid supplements this study has found NTDs that lead to HCP. This finding, that is, the association of taking folic acid nutritional supplements and the development of HCP is interesting considering the clear relationship between folic acid deficiency and the development of NTDs. This is similar to the study from Nigeria that suggested the high incidence of NTDs was due to the lack of use of folic acid by the majority of the mothers of the affected children [16]. Similar studies have suggested the consumption of adequate amounts of folic acid by women before pregnancy and during early pregnancy decreases their risk for having a pregnancy affected by NTDs [23], and a study in Northern China that found the supplementation of folic acid reduced the incidence of HCP and NTDs [15]. An association between advanced maternal age and the development of HCP was not found similar to a study in Mississippi [22]. An increased risk of having a child with HCP among young mothers has been reported [21], however, this study was not designed to determine the mechanism by which birth defects occur among different age groups of mothers. Further investigation is needed to understand the effects of maternal age on HCP and other birth defects. Although this study found additional family members, especially first-degree relatives with HCP similar to studies in Mississippi [22] and Denmark [24], it did not find any association. Familial aggregation and both the genetic and maternal effects play important roles in congenital HCP pathogenesis. However, this study did not investigate genetics as a risk factor for HCP due to the lack of genetic laboratories and clinics; further exploration is recommended. The lack of association in the above variables may be due to limited sample size, constrained resources at the time of data collection while conducting the prospective study, and lack of patient’s clinical data that reduced the number of study variables in the retrospective study.

## Strengths and limitations of the study

The strength of this study was; cross-sectional facility-based study design employed across the four selected diagnostic centers allowed this study to be representative of the study population and the limitation of this study was; the limited amount of time and resource the prospective study had and failure of the MRI machine during this data collection period, as a result, has decreased the total studied population.

## Conclusions

The high prevalence of pediatric HCP observed in both study periods was due to Aqueductal stenosis followed by NTDs with minimal contribution from infectious causes and post-traumatic complications. Non-communicating HCP was the most predominant type of HCP. Infants aged younger than 24 months were a major risk factor in the development of HCP. There is a high association between the amount of folic acid consumption in early pregnancy and the incidence of NTDs which leads to developing HCP. The majority of HCP diagnosed patients had first-degree relatives with congenital HCP.

## Recommendations

It would be ideal if public teaching hospitals affiliated with universities, public and private medical schools, and diagnostic centers across the country conducted similar research regarding the prevalence and risk factors of HCP. This would help in formulating a guideline that can be used across the nation and better understand its public health impact. Further investigation is recommended to understand maternal risk factors associated with the occurrence of congenital and acquired HCP. If a centrally managed database of patients’ clinical history among public and private health institutes was organized under the Ministry of Health, it would help keep records for better management of the patient and will also serve as a medical and public health policy input.

## Data Availability

The dataset used and analyzed during the current study is available from the corresponding author on reasonable request.

## Abbreviations

HCP: Hydrocephalus
MRI: Magnetic Resonance Imaging
CT: Computed Tomography
CSF: Cerebrospinal fluid
MMC: Mennigomyocele
NTD: Neural Tube Defects

## References

[1] Oi S, Takahashi S, Macías IS, Oi S, Miyajima M, Qureshi M, Inagaki T, Rpmero MA, Ma J, Ohira T. Journal of Hydrocephalus. 2009.

[2] Kahle KT, Kulkarni AV, Limbrick DD, Warf BC (2016). Hydrocephalus in children. Lancet.

[3] Chi J (2005). Time trends and demographics of deaths from congenital hydrocephalus in children in the US: National Center for Health Statistics data, 1979 to 1998. J Neurosurg.

[4] Warf BC, Collaboration EANR (2010). Pediatric hydrocephalus in East Africa: prevalence, causes, treatments, and strategies for the future. World Neurosurg.

[5] Barthélemy EJ, Valtis YK, Cochran MF, Martineau L, Park K, Mendel JB (2018). Patterns of Hydrocephalus in Rural Haiti: A Computed Tomography–Based Study. World Neurosurg.

[6] Laeke T, Tirsit A, Biluts H, Murali D, Wester K (2017). Pediatric hydrocephalus in Ethiopia: treatment failures and infections: a hospital-based, retrospective study. World Neurosurg.

[7] Kinasha A, Kahamba J, Semali I (2005). Complications of ventriculoperitoneal shunts in children in Dar Es Salaam. East Central Afr J Surg.

[8] Komolafe EO, Adeolu AA, Komolafe MA (2008). Treatment of cerebrospinal fluid shunting complications in a Nigerian neurosurgery programme. Pediatr Neurosurg.

[9] Lane JD, Mugamba J, Ssenyonga P, Warf BC (2014). Effectiveness of the Bactiseal universal shunt for reducing shunt infection in a sub-Saharan African context: a retrospective cohort study in 160 Ugandan children. J Neurosurg Pediatr.

[10] Neurosurgery TIGtP (2020). Normal CSF Circulation.

[11] Disorders NOoR (2020). Hydrocephalus.

[12] Garne E, Loane M, Addor MC, Boyd PA, Barisic I, Dolk H (2010). Congenital hydrocephalus—prevalence, prenatal diagnosis and outcome of pregnancy in four European regions. Eur J Paediatr Neurol.

[13] Munch TN, Rostgaard K, Rasmussen M-LH, Wohlfahrt J, Juhler M, Melbye M (2012). Familial aggregation of congenital hydrocephalus in a nationwide cohort. Brain..

[14] Jeng S, Gupta N, Wrensch M, Zhao S, Wu YW (2011). Prevalence of congenital hydrocephalus in California, 1991-2000. Pediatr Neurol.

[15] Liu J, Jin L, Li Z, Zhang Y, Zhang L, Wang L (2018). Prevalence and trend of isolated and complicated congenital hydrocephalus and preventive effect of folic acid in northern China, 2005–2015. Metab Brain Dis.

[16] Eke CB, Uche EO, Chinawa JM, Obi IE, Obu HA, Ibekwe RC (2016). Epidemiology of congenital anomalies of the central nervous system in children in Enugu, Nigeria: a retrospective study. Ann Afr Med.

[17] Santos MM, Rubagumya DK, Dominic I, Brighton A, Colombe S, O’Donnell P (2017). Infant hydrocephalus in sub-Saharan Africa: the reality on the Tanzanian side of the lake. J Neurosurg Pediatr.

[18] Gathura E, Poenaru D, Bransford R, Albright AL (2010). Outcomes of ventriculoperitoneal shunt insertion in sub-Saharan Africa. J Neurosurg Pediatr.

[19] Adeloye A (2001). Management of infantile hydrocephalus in Central Africa. Trop Dr.

[20] Awad ME (1992). Infantile hydrocephalus in the south-western region of Saudi Arabia. Ann Trop Paediatr.

[21] Reefhuis J, Honein MA (2004). Maternal age and non-chromosomal birth defects, Atlanta—1968–2000: teenager or thirty-something, who is at risk?. Birth Defects Res A Clin Mol Teratol.

[22] Van Landingham M, Nguyen TV, Roberts A, Parent AD, Zhang J (2009). Risk factors of congenital hydrocephalus: a 10 year retrospective study. J Neurol Neurosurg Psychiatry.

[23] Wald N, Law M, Morris J, Wald D (2001). Quantifying the effect of folic acid. Lancet.

[24] Munch T, Rasmussen M, Wohlfahrt J, Juhler M, Melbye M. Risk factors for primary congenital hydrocephalus-a nationwide cohort study. Am J Epidemiol. 2012;175:S57. Oxford Univ Press Inc Journals Dept, 2001 Evans Rd, Cary, NC 27513 USA.

